# New Compact Antenna Array for MIMO Internet of Things Applications

**DOI:** 10.3390/mi13091481

**Published:** 2022-09-06

**Authors:** Wazie M. Abdulkawi, Mohammed A. Alqaisei, Abdel-Fattah A. Sheta, Ibrahim Elshafiey

**Affiliations:** 1 Electrical Engineering Department, King Saud University, Riyadh 11421, Saudi Arabia; 2 Department of Electrical Engineering, College of Engineering in Wadi Addawasir, Prince Sattam bin Abdulaziz University, Wadi Addawasir 11991, Saudi Arabia

**Keywords:** Internet of Things (IoT), regional area network, multiple-input multiple-output (MIMO) antenna, channel capacity loss (CCL), MVDR beamforming, DOA estimation

## Abstract

A communication system is proposed for the Internet of Things (IoT) applications in desert areas with extended coverage of regional area network requirements. The system implements a developed six-element array that operates at a 2.45 GHz frequency band and is optimized to reduce the size and limit element coupling to less than −20 dB. Analysis of the proposed system involves a multiple-input multiple-output (MIMO) operation to obtain the diversity gain and spectral efficiency. In addition, the radiation efficiency of the proposed antenna is greater than 65% in the operation bandwidth (more than 30 MHz) with a peak of 73% at 2.45 GHz. Moreover, an adaptive beamforming system is presented based on monitoring the direction of arrival (DOA) of various signals using the root MUSIC algorithm and utilizing the DOA data in a minimum variance distortionless response (MVDR) technique beamformer. The developed array is found to have an envelope correlation coefficient (ECC) value of less than 0.013, mean effective gain (MEG) of more than 1 dB, diversity gain of more than 9.9 dB, and channel capacity loss (CCL) of less than 0.4 bits/s/Hz over the operation bandwidth. Adaptive beamforming is used to suppress interference and enhance the signal-to-interference noise ratio (SINR) and is found to achieve a data rate of more than 50 kbps for a coverage distance of up to 100 km with limited power signals.

## 1. Introduction

The Internet of Things (IoT) provides a paradigm of physical items equipped with software tools to achieve efficient data exchange among various devices. The challenges of IoT systems depend on the nature of the application. In applications related to rural and desert areas, the system typically involves sensing units that are distributed over large areas. Being operated with batteries or solar energy sources, the units should limit power use to reduce maintenance costs. The system also connects to a node that is connected to a cellular network, which can be tens of kilometers away, to make acquired data available on the internet to monitoring and control centers [1,2]. In [3,4] the monopole UWB antennas for IoT applications were proposed and designed with an inside cut-feed structure and linearly tapered transmission line, respectively.

IoT systems thus depend on a high level of intelligence to optimize system performance in a challenging environment. Part of the system intelligence can be achieved by using efficient and adaptive beamforming capability associated with the use of multiple-input multiple-output (MIMO) antenna arrays [5,6,7,8]. MIMO systems offer various advantages, including an increase in the data rate, enhancement of spectral efficiency, and boosting the diversity gain [9,10,11]. These systems provide higher capacity gain and are more robust to noise and fading channel conditions, allowing for larger data rates without sacrificing additional airwaves or transmission power [12,13].

The critical issues when building an antenna array for MIMO systems include a small distance between the antenna elements, compact size, low ECC, high isolation, and high efficiency [14,15,16,17]. Higher channel capacity can be accomplished by increasing the number of antenna elements [18]. The MIMO antenna elements are placed very close which causes mutual coupling which means reducing the MIMO system performance. As a result, the mutual coupling between the antenna elements remains the core issue [19,20]. Various decoupling approaches are presented and investigated in the literature to improve isolation [21,22].

In [23], 2 × 2 and 4 × 4 MIMO antenna systems were reported with wideband characteristics. The reported MIMO antennas were decoupled using the neutralization technique, in which a neutralization line is connected between the adjacent elements. This resulted in the isolation of < −22 dB and < −23 dB for 2 × 2 and 4 × 4, respectively. Similarly in [24], the dummy elements-decoupling isolation technique provided isolation of < −20 dB between the adjacent antenna elements. However, the size of the reported antenna was relatively large which was 44 mm. The reported design can enhance mutual coupling of the antenna, which boosts and enhance the performance efficiency of the antenna. In [25], a dual-band 4 × 4 MIMO antenna system was proposed in which the self-decoupled antenna technique was used. The proposed MIMO antenna system operated on 3.4–3.6 GHz and 4.8–5.0 GHz with isolation better than −17 dB for the low band and −20 dB for the high band. A multi-slot decoupling technique was proposed [26], in which a dual-band MIMO antenna system was designed. This system operated on 3.4–3.8 GHz and 4.8–5.0 GHz, giving isolation of −15.5 dB and −19 dB (−6 dB), respectively. The multi-slot structure is introduced to reduce mutual coupling between the antenna elements. In the reported work [27], an eight-element MIMO antenna array was proposed to cover 2.4–3.6 GHz bandwidth. The microstrip feeding-line decoupling technique was applied which gives −15 dB isolation and better channel capacity.

MIMO and beamforming techniques are the key enablers of enhanced connectivity and increased data rate. The beamforming technology is used to divert the radiations towards the desired direction and avoid them in unwanted directions. In other words, beamforming is used to focus the beam in the desired direction and cancel the undesirable signals and reduce the side lobe effect [28,29].

This paper proposes a MIMO-slotted square-patch antenna array, that was developed to be low-cost and small enough to be used in IoT applications and devices. This antenna can cover some IoT wireless standards, such as Bluetooth, Bluetooth smart (BLE), WiFi, and ZigBee.

The array has a reconfigurable architecture that allows it to operate in either receive or broadcast mode at the desired frequency.

The proposed antenna configuration is designed to operate in the industrial science medical band (ISM-band). Two prototypes are presented to be adopted for IoT applications at 2.45 GHz. The first configuration has only one element of the slotted square-patch antenna, however the second one has a six-element array of the proposed antenna. The single and MIMO antennas are designed, fabricated, and measured. The measured results correlate well with the simulated ones. Each antenna operates in the desired frequency band of operation with the minimum acceptable isolation of (≤ −20 dB) and ECC less than 0.2 for any two antennas of the array. Since the proposed solution can have a compact and low profile antenna structure, which has very good isolation and ECC value, it is very promising for practical IoT MIMO applications. After that, the beamforming function is analyzed based on the proposed array to show the potential of the developed array in enhancing signal-to-interference noise ratio (SINR), by suppressing unwanted interference.

In addition, the paper provides a simulation analysis of the proposed antenna in the typical IoT communication system to investigate the diversity gain (DG), capacity, and beamforming capabilities.

The study is scheduled as follows. A single element of the slotted square patch antenna is discussed in Section 2. In Section 3, the antenna array is designed and explained. The array parameters and MIMO performance are discussed in Section 4 and Section 5. The beamforming capabilities of the proposed array are shown in Section 6. In Section 7, the performance of the proposed communication system in the regional area network is discussed. The conclusions and discussions are presented in Section 8.

## 2. Single Element Antenna

**Figure 1:**
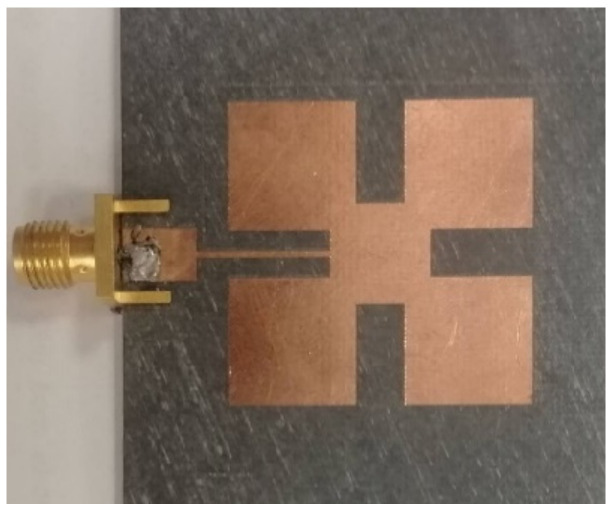
Single antenna element (Ant 1). The patch dimensions are optimized to operate at the 2.45 GHz frequency as: patch length and width = 28 mm, slot length = 9.3 mm, and slot width = 4.5 mm.

Figure 1 illustrates the proposed slotted square-patch antenna (Ant 1). The antenna is designed as a microstrip square patch, along with etched four slots along the *X*- and *Y*-axes. All of the slots are of the same length and width [30]. The slotted square patch can be designed based on the fractal concept [31,32,33,34] or TM_mn0_ modes [35,36]. The latter is adopted and considered as a square cavity with magnetic walls. The field inside the square cavity matches those of the TM^z^_mn0_ modes. The coherent modes (TM^z^_mn0_) and the resonance frequencies of the square-patch type can be calculated using the same way as described in [37,38,39]. In our case, the proposed structure is based on TM_010_ and TM_100_ modes. Only one mode (TM_010_) is excited in the band of interest.

The antenna element is printed on the front surface of an RT Duroid 5880 dielectric substrate with relative permittivity of 2.2 and a loss tangent of 0.0009. The xyz dimensions are 41.5 × 38.5 × 1.57 mm^3^. The antenna is designed and optimized using full-wave electromagnetic computer simulation technology (CST Ver.2021).

**Figure 2:**
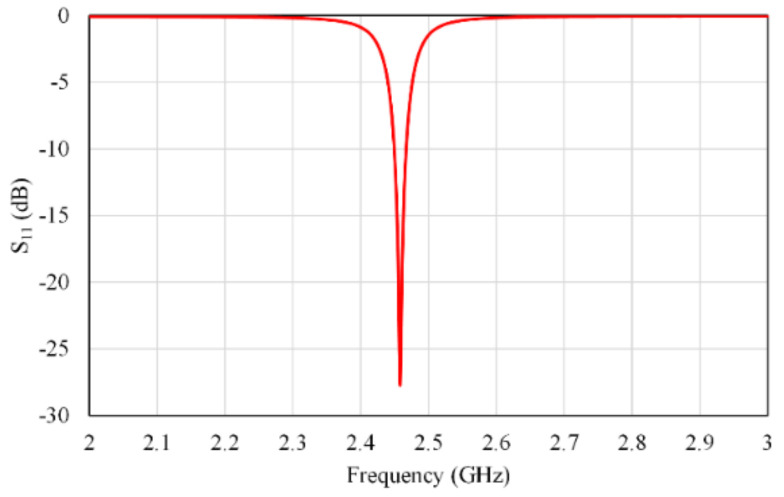
S_11_ response of a single antenna element; patch length and width = 28 mm, slot length = 9.3 mm, and slot width = 4.5 mm.

**Figure 3:**
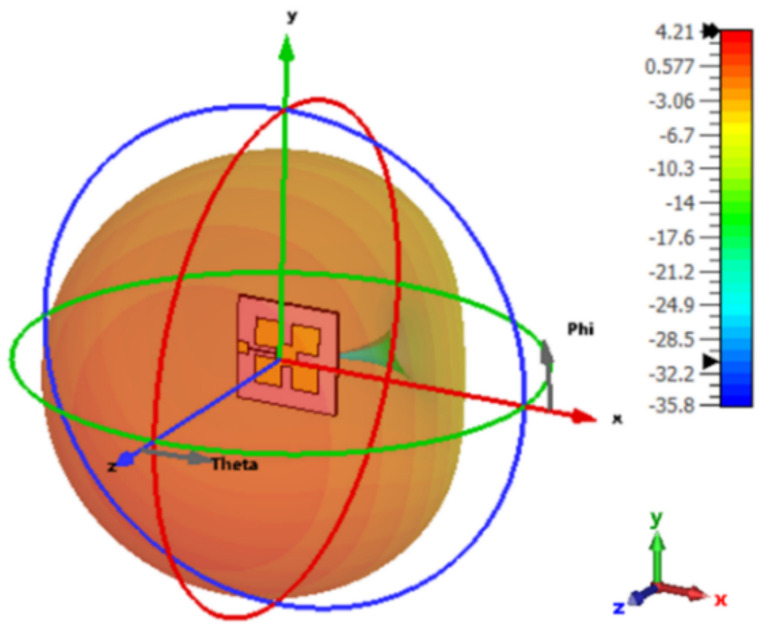
The simulated 3D radiation pattern of Ant 1.

Following the mathematical formulas described in [30,37,38,39], the primary antenna configuration resonating at 2.45 GHz is designed and fabricated. The fabricated prototype antenna is connected to a 50-ohm SMA connecter. The reflection coefficient (S_11_) and radiation pattern at 2.45 GHz of Ant-1 are shown in Figure 2 and Figure 3, respectively.

**Figure 4:**
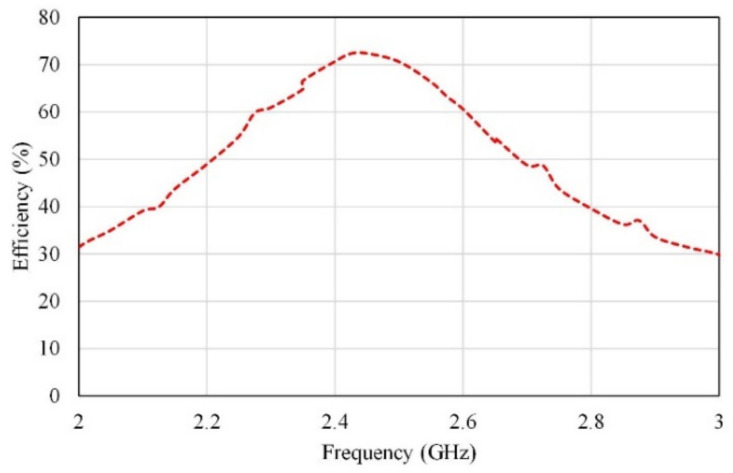
The efficiency of a single antenna element.

Figure 4 shows the efficiency of the proposed single antenna. It is observed from this figure that the efficiency is greater than 65% in the operation bandwidth with a peak of 73% at 2.45 GHz.

## 3. Proposed Array Structure

**Figure 5:**
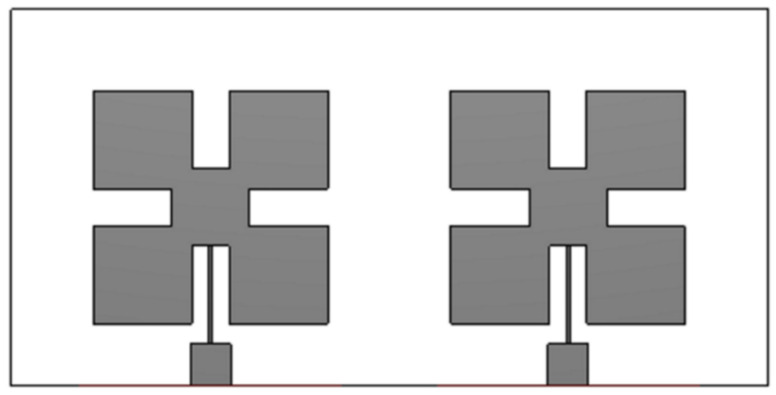
The two-element array of Ant 1.

The proposed design is further processed from a single element to a two-element array, as shown in Figure 5.

**Figure 6:**
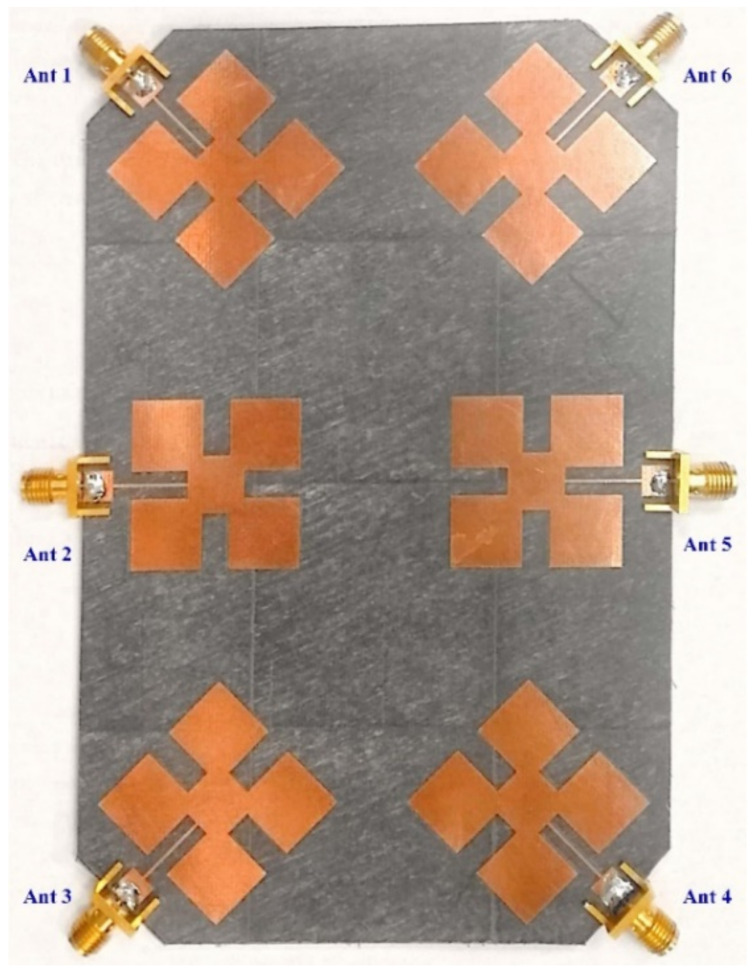
Photograph of the fully grounded proposed MIMO antenna array.

The configuration has proceeded further to develop the three-port and four-port arrays. Finally, a fully grounded six-port array is developed, as shown in Figure 6, where six patch elements are printed on the top side of the Rogers substrate. The overall size of the antenna system is 97 × 150 × 1.6 mm^3^. Each element of the designed MIMO system is excited through a 50 ohm SMA connector.

**Figure 7:**
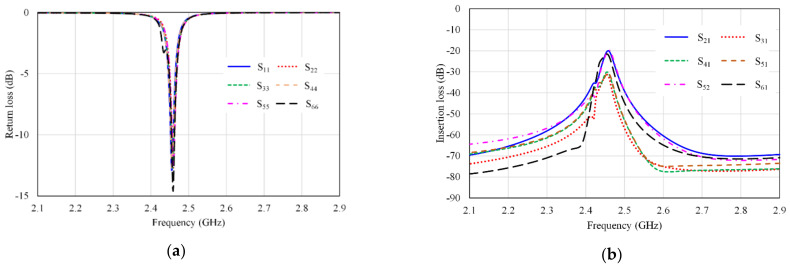
S-parameters for the six-port MIMO antenna array (**a**) return loss (S_ii_), and (**b**) insertion loss (S_ij_).

Increasing the mutual coupling reduces the isolation which adversely affects the performance of the antenna, and therefore it is difficult to achieve a low mutual coupling within a compact size in the design of the MIMO array antenna system [40,41,42]. In the proposed design, we increase the separation between two adjacent antennas in the proposed MIMO to around 25 mm (about a quarter wavelength) to obtain high isolation between the antenna elements. In addition, to achieve compactness, the antennas (Ant 1-Ant 6) are arranged in the manner shown in Figure 6. The simulated S-parameters of the proposed six-port antenna are shown in Figure 7.

In Figure 7a, the reflection coefficients S_ii_ (i = 1, 2, …, 6) are almost identical. It is observed from the results in Figure 7b that the isolations between antenna elements represented by S_ij_ (i = 1, 2, …, 6, and j = 1, 2, …, 6, I ≠ j) are less than −20 dB. Therefore, the designed antenna has better isolation of more than 20 dB.

Because of symmetry, the following S-parameters similarities are remarked:

S_11_ = S_22_ = S_33_ = S_44_ = S_55_ = S_66_, S_21_ = S_32_ = S_54_ = S_65_, S_31_ = S_64_, S_41_ = S_63_, S_51_ = S_53_ = S_62_ = S_42_, S_61_ = S_43_ (1)

## 4. Array Parameters

The performance of the proposed six-element antenna array is shown and discussed in this section.

### 4.1. S-Parameters

**Figure 8:**
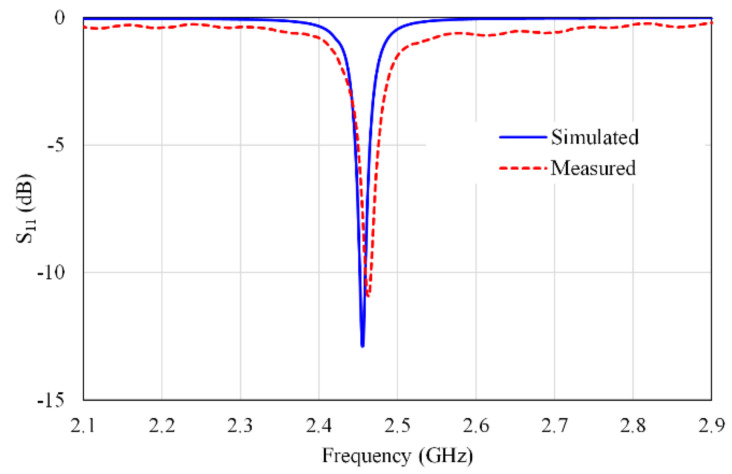
Simulated and measured S_11_.

The simulated S-parameters for the proposed MIMO antenna are previously shown in Figure 7. Figure 8 illustrates the simulated and measured reflection coefficient of Ant 1.

**Figure 9:**
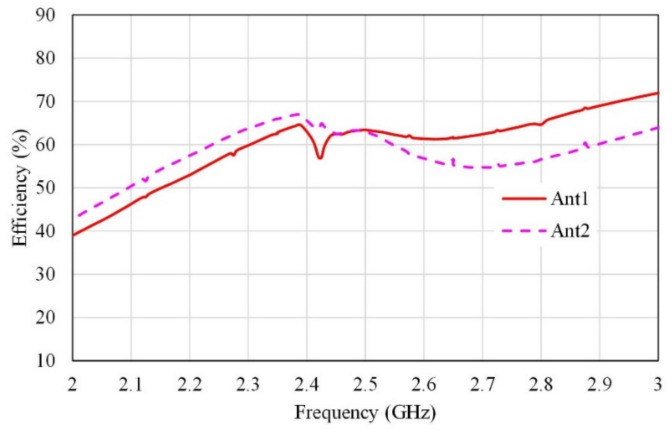
MIMO antenna radiation efficiency.

The reflection coefficients of other antennas (S_22_, S_33_, S_44_, S_55_, and S_66_) are in close agreement with the results presented in Figure 8. Figure 9 shows the efficiency of the proposed MIMO antenna. It is observed from this figure, that the efficiencies of Ant 1, Ant 3, Ant 4, and Ant 6 are about 57–63%, however the efficiencies of Ant 2 and Ant 5 are about 62–65% in the operation bandwidth.

**Figure 10:**
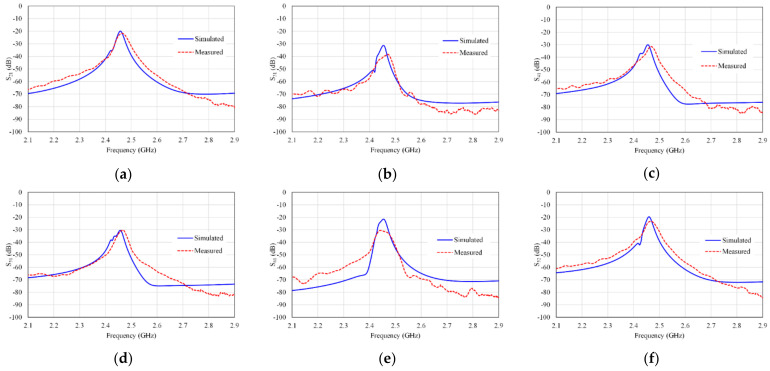
Simulated and measured S parameters (**a**) S_21_, (**b**) S_31_, (**c**) S_41_, (**d**) S_51_, (**e**) S_61_, and (**f**) S_52_.

The simulated and measured isolations of these stated antennas (S_21_, S_31_, S_41_, S_51_, S_61_, and S_52_) are shown in Figure 10. Thus, high isolations between antennas are obtained, which are better than 20 dB overall.

### 4.2. Radiation Performances

**Figure 11:**
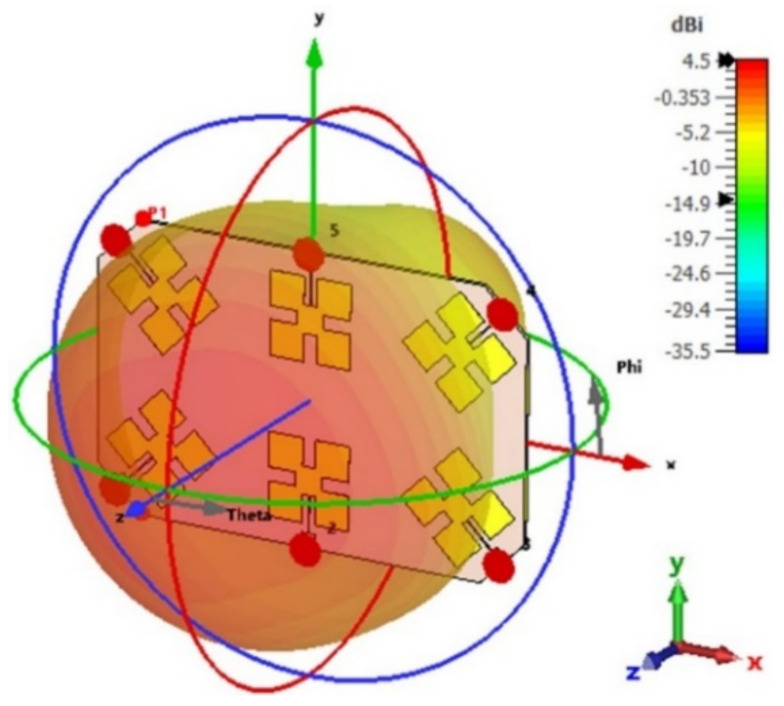
The simulated 3D radiation pattern of Ant 1, Ant 3, Ant 4, and Ant 6 at 2.45 GHz.

**Figure 12:**
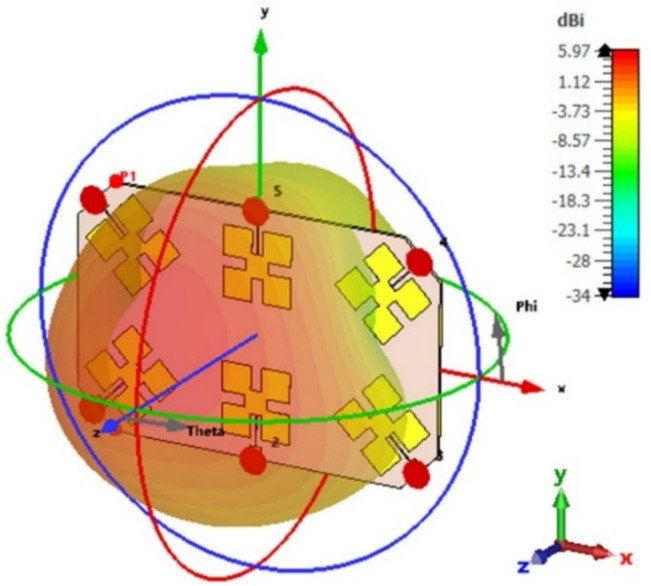
The simulated 3D radiation pattern of Ant 2, and Ant 5 at 2.45 GHz.

The radiation patterns of the presented array antenna are analyzed and measured. Figure 11 and Figure 12 show the simulated far-field radiation patterns of a six-port MIMO array.

The gain of a single element is 4.21 dB as given in Figure 3 for the short ground plane. For the array, the gain becomes 4.5 dB for Ant 1, Ant 3, Ant 4, and Ant 6 (as given in Figure 11) and 5.97 dB for Ant 2 and Ant 5 (as given in Figure 12). This is expected since in the array configuration, the ground plane is large and most of the radiation comes from the front side.

**Figure 13:**
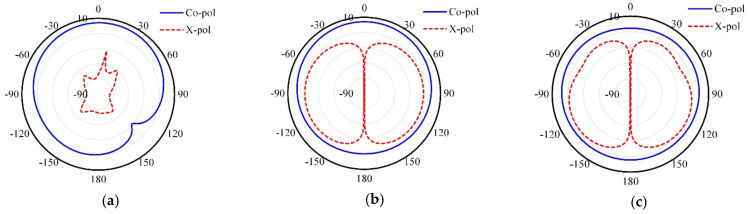
Co-polarized and cross-polarized radiation patterns of the proposed antenna (**a**) xz-plane, (**b**) yz-plane, and (**c**) xy-plane.

Figure 13 shows the co- and cross-polarized radiation patterns for the suggested antenna at 2.45 GHz on the xz-plane, yz-plane, and xy-plane, respectively.

**Figure 14:**
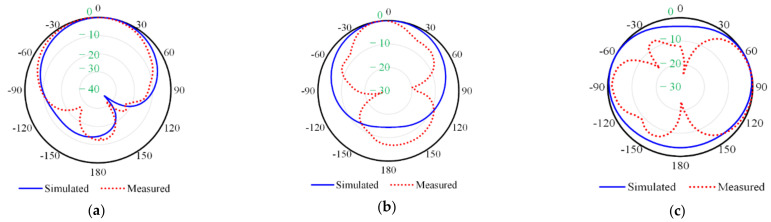
The simulated and measured radiation pattern of the proposed antenna at 2.45 GHz on the (**a**) xz-plane, (**b**) yz-plane, and (**c**) xy-plane.

The normalized 2D radiation patterns for the fabricated prototype are measured and compared with the simulated ones, as shown in Figure 14.

Figure 14 shows the simulated and measured radiation patterns of the six-port MIMO antenna at 2.45 GHz on the xz-plane, yz-plane, and xy-plane, respectively.

To measure the radiation patterns for the proposed antenna, the antenna measurement system GEOZONDAS in a time domain (GEOZONDAS-TDAMS) is used [43]. A measurement setup was established to validate the far-field radiation pattern of the antenna. The antenna under test was placed on the top of the rotor.

**Figure 15:**
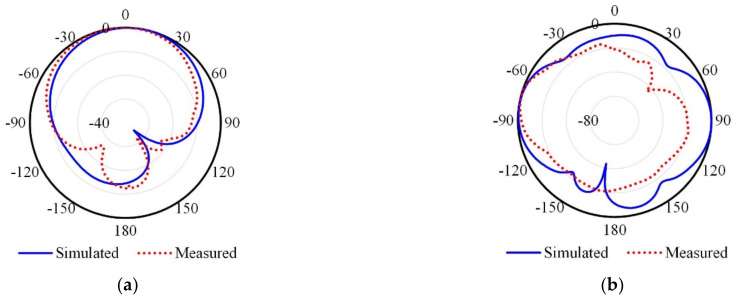
Radiation patterns of the proposed MIMO antenna in the xz-plane, (**a**) co-polarized and (**b**) cross-polarized.

The results in Figure 15 illustrate the co-polarized (co-pol) and cross-polarized (x-pol) radiation patterns for the proposed MIMO antenna in the xz-plane.

## 5. MIMO Performance Parameters

To evaluate the potential MIMO performance of the proposed antenna array, the envelope correlation coefficient (ECC), the diversity gain (DG), the mean effective gain (MEG), and the channel capacity loss (CCL) are measured and analyzed.

The ECC parameter is used to measure the performance of the MIMO antenna system and it can be calculated using the S parameter method by the following equation [44,45]:

(2) $$\rho_{ij}=\frac{{\left|S_{ii}^{*}S_{ij}+S_{ji}^{*}S_{jj}\right|}^{2}}{\left(1-{\left|S_{ii}\right|}^{2}-S_{ij}^{2}\right)\left(1-{\left|S_{ji}\right|}^{2}-S_{jj}^{2}\right)}$$

where *i* = 1, 2, 3, 4, 5, 6 and *j* = *i* + 1, *i* + 2, …, 6. In our design, the 15 values of ECC are calculated, which are; *ρ*_12_, *ρ*_13_, *ρ*_14_, *ρ*_15_, *ρ*_16_, *ρ*_23_, *ρ*_24_, *ρ*_25_, *ρ*_26_, *ρ*_34_, *ρ*_35_, *ρ*_36_, *ρ*_45_, *ρ*_46_, and *ρ*_56_. The spectral efficiency of the MIMO antenna system suffers when the ECC becomes high. It means that the lower value of ECC is preferable [9]. When the value of *ρ* approaches 0, a MIMO antenna system exhibits near-perfect performance [46].

In addition, the value of ECC can be calculated by using the radiation pattern method which is more accurate than the S parameter method. The ECC between Ant *i* and Ant *j* can be calculated using their far-field radiation patterns with the following equation [47,48]:

(3) $$\rho_{ij}=\frac{{\left|\int \int_{4\pi }\bar{F_{i}}\left(\theta ,\varphi \right)\cdot {\bar{F_{j}}}^{*}\left(\theta ,\varphi \right)d\Omega \right|}^{2}}{\int \int_{4\pi }{\left|\bar{F_{i}}\left(\theta ,\varphi \right)\right|}^{2}d\Omega \cdot \int \int_{4\pi }{\left|\bar{F_{j}}\left(\theta ,\varphi \right)\right|}^{2}d\Omega }$$

**Figure 16:**
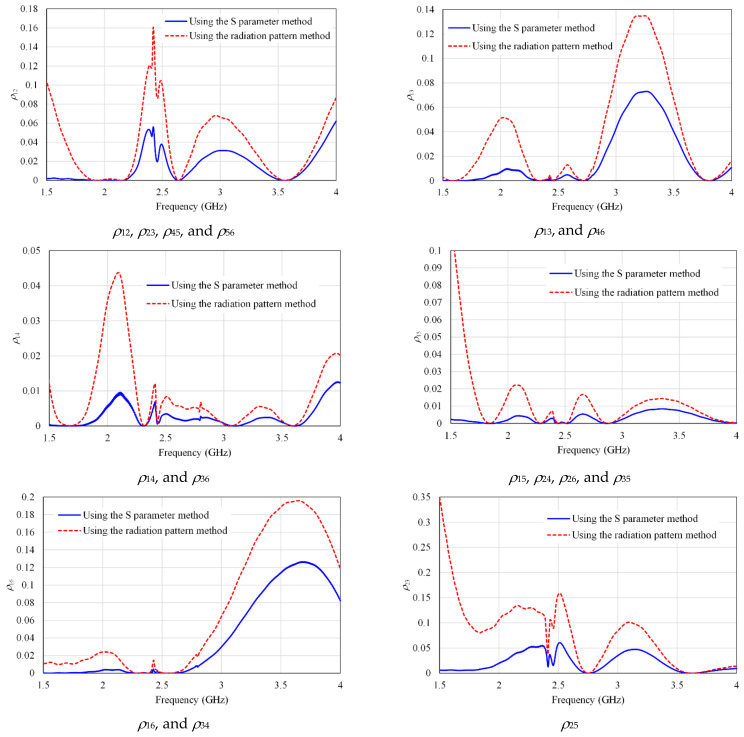
The ECC of the proposed MIMO antenna using the S parameter and radiation pattern methods.

Figure 16 displays the result of the ECC curves, which are calculated from both the S-parameter and the radiation pattern methods.

It is observed from this figure that the maximum values of ECC have occurred between Ant 1 and Ant 2 (*ρ*_12_), Ant 2 and Ant 3 (*ρ*_23_), Ant 4 and Ant 5 (*ρ*_45_), and Ant 5 and Ant 6 (*ρ*_56_). The maximum value of ECC is about 0.16.

An estimate of DG performance depends on measuring the transmission power loss with a value of it set to 10 dB [49,50]. The DG can be computed by:

(4) $${\mathrm{DG}}_{ij}=10\;\sqrt{1-{\left|\rho_{ij}\right|}^{2}}$$

**Figure 17:**
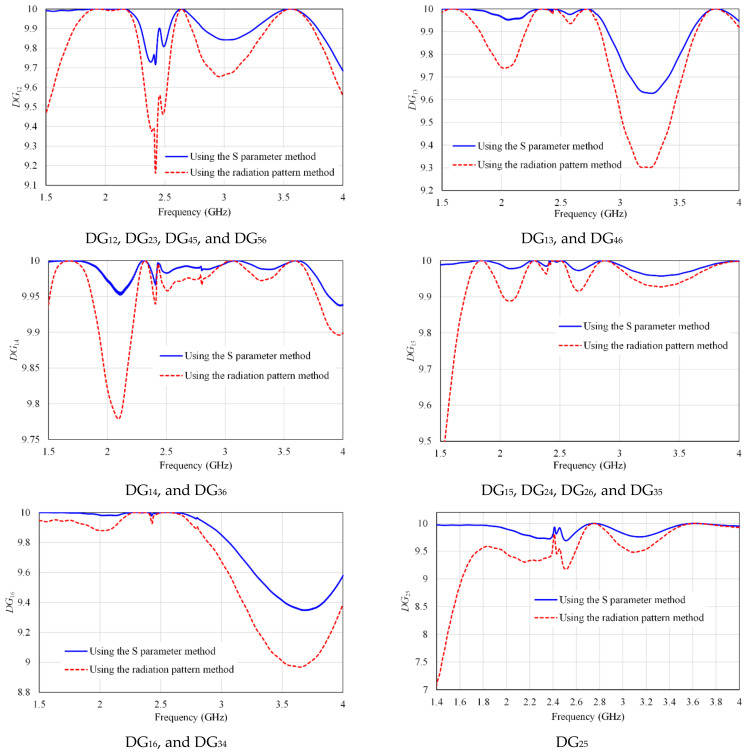
The DG of the proposed MIMO antenna by using the S parameter and radiation pattern methods.

Figure 17 shows the DG curves of our array, which are near 10 dB in the operation bandwidth. It means the proposed MIMO antenna has a good diversity performance.

**Figure 18:**
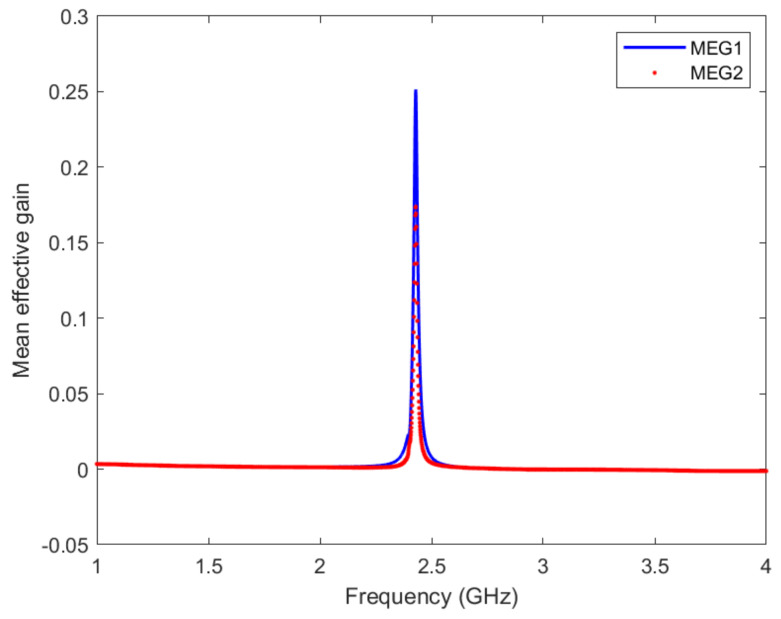
The MEG of the proposed MIMO antenna. (MEG1) is when Ant 1, Ant 3, Ant 4, or Ant 6 is under observation, however, the second curve (MEG2) is when Ant 2 or Ant 5 is under observation.

The MEG is another essential parameter used to measure the diversity performance of the MIMO antenna. It is the ratio between the mean powers received by diversity gain to the mean power received by an isotropic antenna. The MEG can be measured using the following equation [51,52]:

(5) $${\mathrm{MEG}}_{i}=0.5\eta_{iRad}=0.5\left(1-\overset{6}{\underset{j=1}{\sum }}{\left|S_{ij}\right|}^{2}\right)$$

where *i* is the active gain and $\eta_{iRad}$ is the radiation efficiency of this antenna. In our design, two-MEG curves are calculated from the S-parameters, as shown in Figure 18. The first one (MEG1) is when Ant 1, Ant 3, Ant 4, or Ant 6 is under observation, however, the second curve (MEG2) is when Ant 2 or Ant 5 is under observation.

It is observed from this figure that the maximum values of MEGs have occurred when Ant 1, Ant 3, Ant 4, or Ant 6 is under observation. The maximum value of MEG is about 0.25.

The CCL of the MIMO antenna is also one of the most important parameters to measure diversity performance. It is used to define the maximum achievable limit of the information transmission rate, and it can be calculated using [46]:

(6) $$\mathrm{CCL}=-\log_{2}\det\left(\alpha \right)$$

where

(7) $$\alpha =\left[\begin{array}{cccccc} \alpha_{12} & \alpha_{13} & \alpha_{14} & \alpha_{14} & \alpha_{15} & \alpha_{16}\\ \alpha_{21} & \alpha_{22} & \alpha_{23} & \alpha_{24} & \alpha_{25} & \alpha_{26}\\ \alpha_{31} & \alpha_{32} & \alpha_{33} & \alpha_{34} & \alpha_{35} & \alpha_{36}\\ \alpha_{41} & \alpha_{42} & \alpha_{43} & \alpha_{44} & \alpha_{45} & \alpha_{46}\\ \alpha_{51} & \alpha_{52} & \alpha_{53} & \alpha_{54} & \alpha_{55} & \alpha_{56}\\ \alpha_{61} & \alpha_{62} & \alpha_{63} & \alpha_{64} & \alpha_{65} & \alpha_{66} \end{array}\right]$$

and

(8) $$\alpha_{ii}=1-\overset{6}{\underset{j=1}{\sum }}{\left|S_{ij}\right|}^{2}$$

(9) $$\alpha_{ij}=-\left(S_{ii}^{*}S_{ij}+S_{ji}^{*}S_{jj}\right)$$

**Figure 19:**
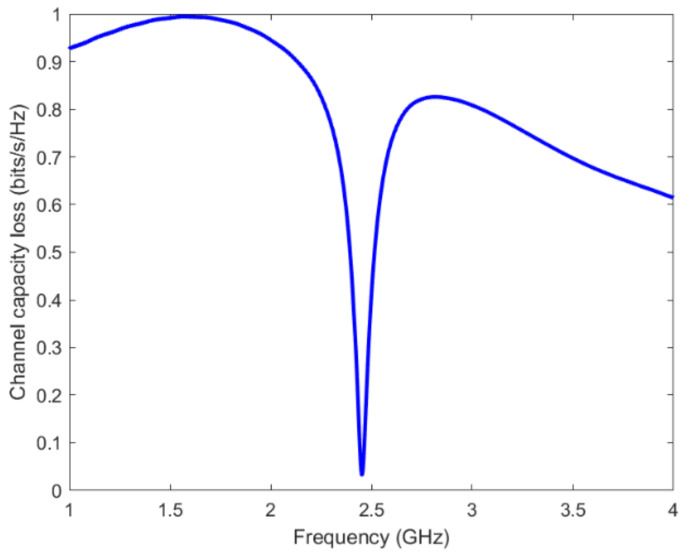
The CCL of the proposed MIMO antenna.

The computed values of CCL for the proposed antenna are shown in Figure 19. It is observed that the CCL is almost below the standard value of 0.4 bits/s/Hz for a practical MIMO antenna system in the operation bandwidth.

Then, we use our antenna parameters to show how the antenna mutual coupling affects the performance of transmission over a MIMO channel. For the DG estimation, the communication system is set to have a transmitter of one element, while the receiver has a six-elements array. QPSK modulation is conducted over a 1 × 6 quasi-static frequency-flat Rayleigh channel [53,54,55]. The simulation is conducted using MATLAB, and the channel path correlation and element coupling are taken into consideration. The suggested communication system operates at 2.45 GHz and the simulated SNR range is 0 to 15 dB.

**Figure 20:**
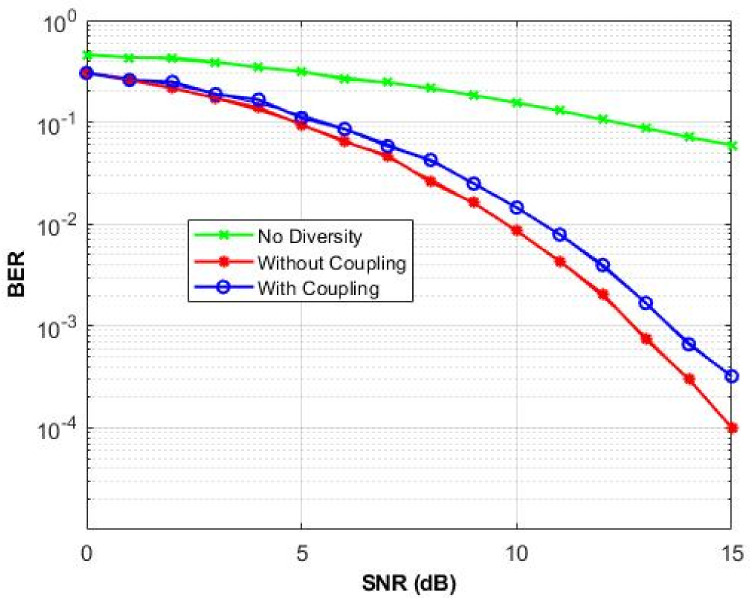
The BER vs. SNR curve.

Figure 20 shows the BER achieved using diversity gain with and without consideration of element coupling. The results are also compared to BER for no diversity gain. As observed from this figure that the diversity gain when BER is equal to 0.01 is about 12 dB.

**Figure 21:**
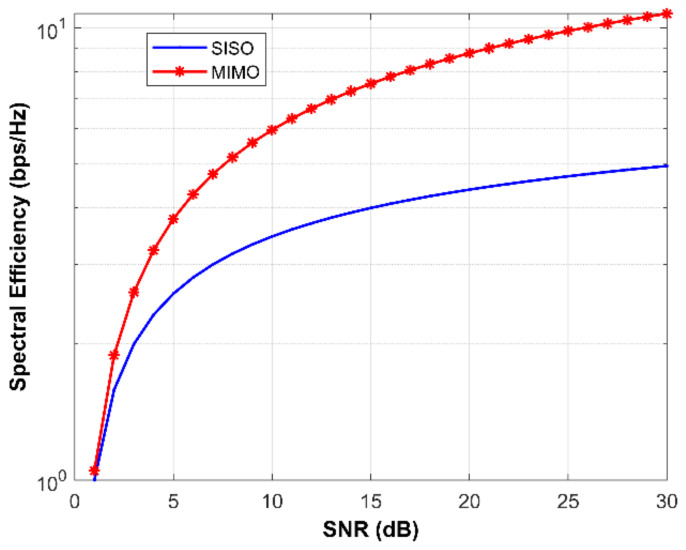
The channel capacity of the proposed MIMO antenna.

To investigate MIMO channel capacity, a simulation is conducted to investigate beam steering to six users, using the six-element proposed array. The spectral efficiency of the MIMO system compared to the SISO system is shown in Figure 21.

Table 1 compares the proposed design with some recently reported 5G terminal MIMO arrays. The table shows how good isolation is achieved in the proposed work. Compared to the recent work, the proposed design shows wider operating bandwidth with very good isolations. In addition, the ECC value is better which makes this design more appropriate in terms of performance and isolation, thus making it highly recommended for IoT mobile applications.

Table 1 shows how the implementation of our suggested structure has the best performance although the six elements are arranged in a compact size with coupling between the antenna array elements <−20 dB.

## 6. Beamforming Function

**Figure 22:**
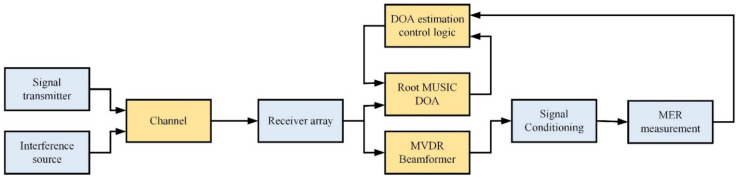
Modeling of Beamforming Receiver.

After developing the MIMO array and testing the antenna parameters, we try to achieve the beamforming functions. Figure 22 shows the block diagram of the modeling of the receiver with beamforming. The simulation is conducted using a SIMULINK environment. The signal transmitter implements a QAM modulator and passes a raised cosine transmit filter. The channel uses the additive white Gaussian noise (AWGN) channel. The receiver is narrowband plane waves incident on the elements of the sensor array.

We use an adaptive beamforming system to suppress the interference signal and thus enhance the SINR. The beamformer depends on implementing the narrowband minimum-variance distortionless response (MVDR) [56]. The beamformer preserves the signal power in the given direction while suppressing interferences from other directions. The system depends on estimating the direction of arrival (DOA) of various signals to localize the direction of desired and interference signals. In particular, we used high-resolution DOA estimation based on the root-MUSIC DOA that is based on the eigenvalue decomposition of the covariance matrix of the receiver sensor array. After identifying the directions of interference and transmitted signals, we use MVDR beamforming to suppress the interference and enhance the desired signal. In addition, adaptive negative feedback EVM is used to accurately follow DOA for both the wanted signal source and the unwanted interference [56].

The model assumes receiving data from *D* uncorrelated or signal sources *s_d_* (*t*). In addition, for an array with *M* elements, the *m*^th^ element data *x_m_* (*t*) includes AWGN n_m_ (*t*). We thus have:

(10) $$x\left(t\right)=As\left(t\right)+n\left(t\right)$$

(11) $$s\left(t\right)=\left[s_{1}\left(t\right),\;s_{2}\left(t\right),\;\ldots ,\;s_{M}\left(t\right)\right]$$

(12) $$A=\left[\left.a\left(\theta_{1}\right)\right|\left.a\left(\theta_{2}\right)\right|\left.\ldots \right|a\left(\theta_{D}\right)\right]$$

where *x*(*t*) is an *M* × 1 vector of a received signal at time *t*.

The *M* × *D* matrix *A* represents the arrival vectors, where each source contributes to one column of *A*. θ_d_ represents azimuth and the elevation angles of the d^th^ source. *S*(*t*) is a D-by-1 vector of source signal values from D sources.

The covariance matrix, *R_x_*, is derived from the received signal data and is associated with the signal covariance matrix *R_s_* and the noise covariance matrix.

(13) $$R_{x}=E\left\{xx^{H}\right\}=AR_{s}A^{H}+\sigma_{n}^{2}I$$

(14) $$R_{s}=E\left\{ss^{H}\right\}$$

Multiple measurements of the sensor data are averaged to obtain $R_{x}$

(15) $$R_{x}=\frac{1}{T}\overset{T}{\underset{k=1}{\sum }}x\left(t\right)x{\left(t\right)}^{H}$$

where *T* is the number of snapshots.

The estimation of the DOA is enhanced through a control logic that makes use of the error vector magnitude (EVM) that is computed from the modulation error ratio (MER).

Three cases are studied in this work which are: case 1: (3 × 2) array with signal angles (azimuthal, elevation) (45°, 45°) and interference angles (azimuthal, elevation) (30°, 90°); case 2: (3 × 2) antenna array with signal angles (30°, 45°) and interference angles (45°, 70°), and case 3: (3 × 4) array using two (3 × 2) arrays, with signal angles (30°, 45°) and interference angles (45°, 70°), respectively.

**Figure 23:**
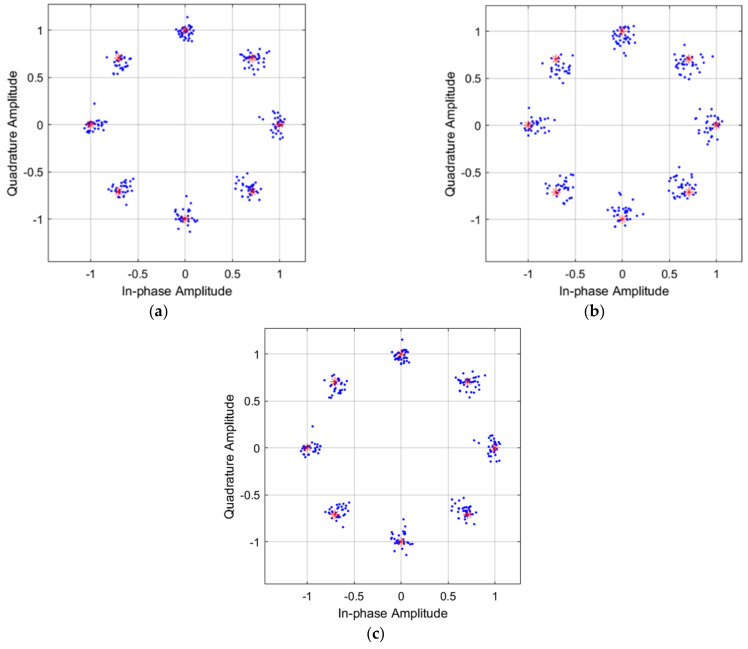
The constellation diagrams of 8 PSK for (**a**) case 1, (**b**) case 2, and (**c**) case 3.

**Figure 24:**
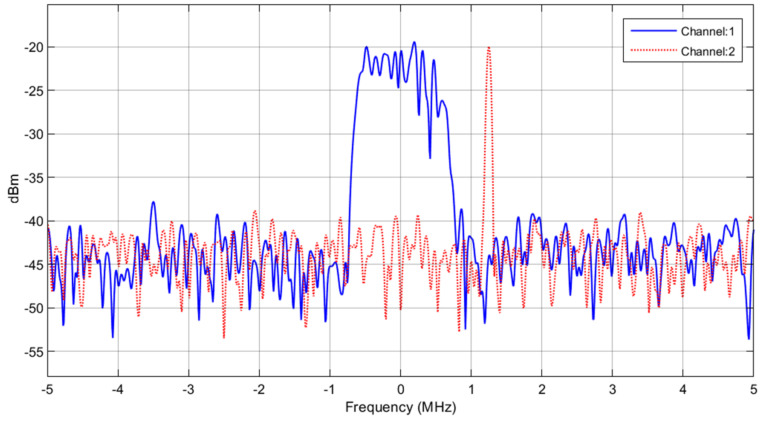
Signal (channel 1) and interference (channel 2) input spectrum of the RF transmitter.

The eight-PSK I/Q amplitude constellation diagrams for case 1, case 2, and case 3 are shown in Figure 23a–c, respectively. The signal and interference input spectrum of the RF transmitter, with a peak power of −20 dBm is shown in Figure 24.

**Figure 25:**
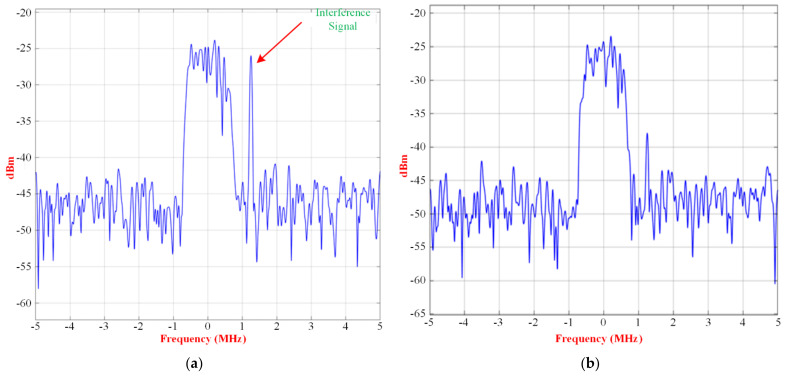
Signal and interference input spectrum of the RF receiver for case 1 (**a**) without beamforming, and after (**b**) with beamforming.

**Figure 26:**
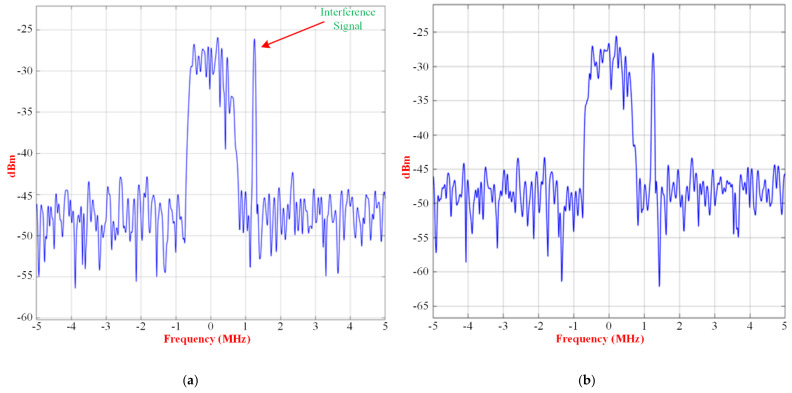
Signal and interference input spectrum of the RF receiver for case 2 (**a**) without beamforming, and (**b**) with beamforming.

**Figure 27:**
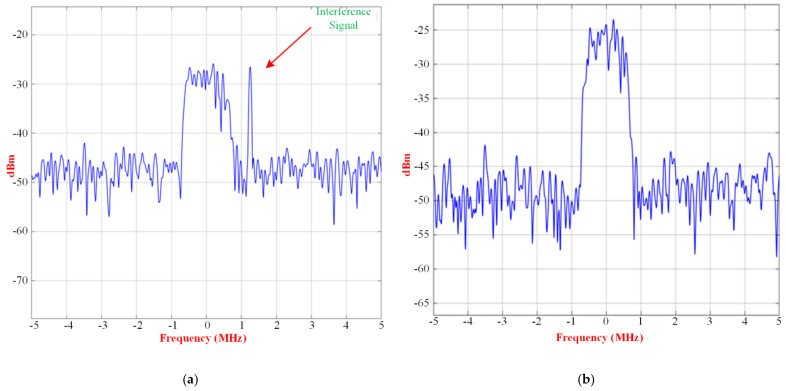
Signal and interference input spectrum of the RF receiver for case 3 (**a**) without beamforming, and (**b**) with beamforming.

The signal and interference input spectrum of the RF receiver, without beamforming and with beamforming, case 1, case 2, and case 3, are shown in Figure 25, Figure 26 and Figure 27, respectively.

There is an improvement of 12 dB of peak power in the interference with the use of beamforming for case 1. For case 2, the interference and signals, which have similar peak power, are close and little interference suppression can be achieved by the beamformer.

However, for the same direction of arrivals, a better resolution is achieved with an array size of 3 × 4 in case 3, and the interference signal is almost removed, compared to the desired signal. However, for the same direction of arrivals, better resolution is achieved with an array size of 3 × 4 in case 3, and the interference signal is almost removed, compared to the desired signal.

**Figure 28:**
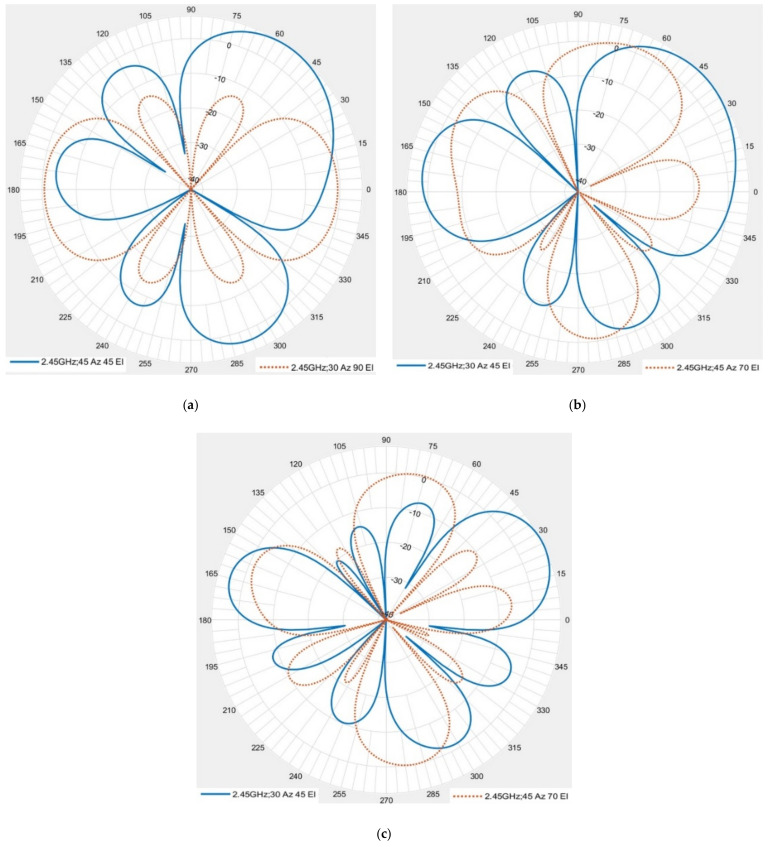
Signal and interference beam-steering for (**a**) case 1 (**b**) case 2 (**c**) case 3.

Figure 28a shows no effect of interference on signal for case 1, as described by the difference between the steered beams to the signal and interference directions. However, when the interference angles are changed to (45°, 70°) and the signal angles are changed to (30°, 45°) (case 2), the beam level of interference becomes larger compared to case 1, and the difference between the interference and signal levels becomes small, as shown in Figure 28b.

To solve this problem, the number of antenna arrays is increased to 4 × 3 (case 3). Figure 28c shows that no effect of the interference on the signal for the interference angles (45°, 70°) and the signal angles (30°, 45°).

**Figure 29:**
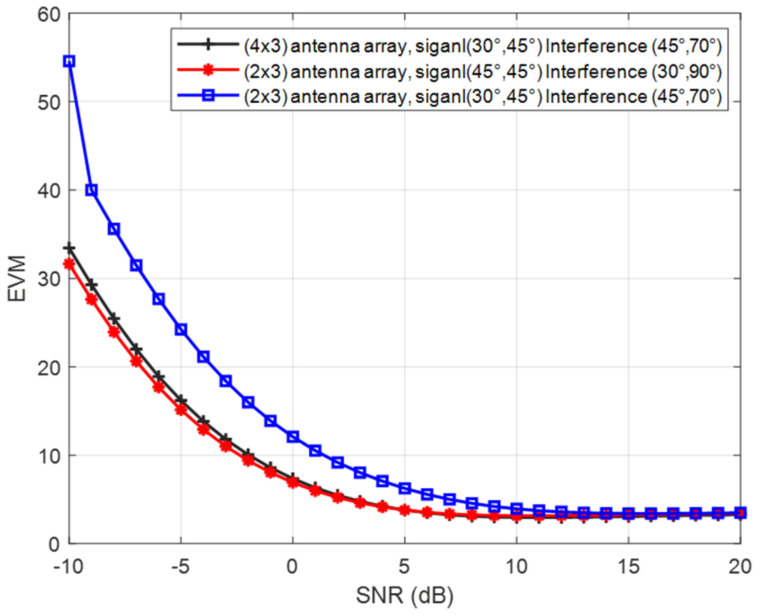
EVM versus SNR over AWGN channels.

The relationship between error vector magnitude (EVM) and signal-to-noise ratio (SNR) is shown in Figure 29. The EVM for case 2 is greater than the other cases by around 22 at −10 dB SNR. This difference reduces exponentially to reach zero at 12 dB SNR. Table 2 summarizes the performance for those three cases.

## 7. Performance in Regional Area Network

The analysis is conducted assuming various schemes with particular channel models and channel coding. In particular, five communication schemes are assumed. The first three schemes assume an additive white Gaussian noise (AWGN) channel with the use of convolution coding with a code rate of 1/2 and soft decoding (1), block coding with a code rate of 3/7 and soft decoding (2); and no channel coding (3). Scheme four assumes a Rician channel with K = 10 dB and a diversity order of 6 and scheme five assumes a Rayleigh fading channel with a diversity order of 6. Using the bit error tool of Matlab, the required bit SNR for an error rate of around 10^−5^ is found to be approximately 4.1, 6.8, 9.5, 10.2, and 13.5 dB for modes 1 to 5, respectively [57,58]. The transmitted power signal, as well as the interference signal, are assumed to be of −10 dBm level each.

**Figure 30:**
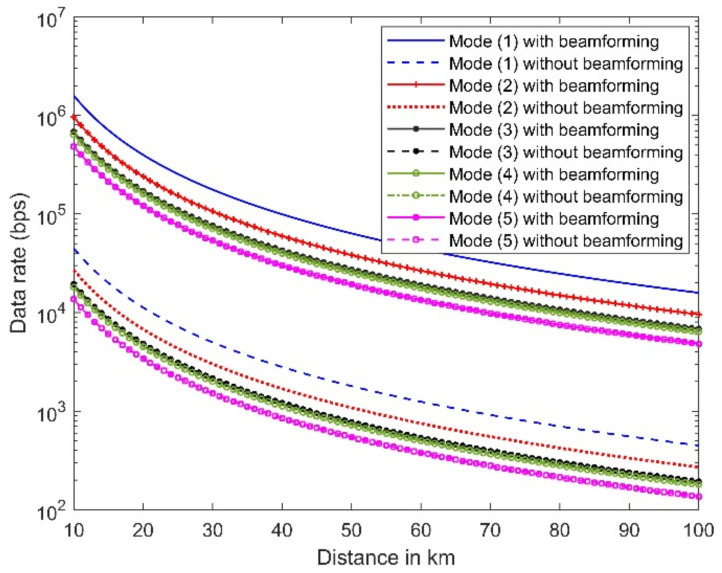
The estimated data rate versus distance corresponds to five communication scheme modes with and without the use of beamforming.

The signal source is assumed to be located at a distance ranging from 10 to 100 km, while the interference level is assumed to be at a much shorter distance of 1 km. For each of the five assumed communication systems schemes, simulations are conducted first using beamforming to suppress the interference signal and then without the use of beamforming. Figure 30 shows the estimated data rate versus the coverage distance. The figure illustrates the importance of beamforming in enhancing the data rate by a ratio of 100.

## 8. Conclusions

A system is proposed for extended coverage of the regional area networks. The system depends on a new compact 3 × 2 array module at 2.45 GHz. Array elements have acceptable isolation of (≤ −20 dB) and ECC is less than 0.2 for any two elements. One or more of these modules can be used to enhance in a central node of the system to collect with other nodes and make use of the array to enhance the signal and reduce unwanted interference levels.

The simulation results illustrate the potential of such a system in providing data rates of acceptable levels for IoT applications for extended coverage networks. Such a system is useful in collecting data in desert areas from widely spaced sensor units. The extended coverage also allows for the connection of the system to a node with cellular coverage to send data through the internet to monitor and control centers. The application examples include collecting environmental data from weather stations and tree-health assessments of palm tree farms.

## Figures and Tables

**Table 1:** Comparison performance.

Ref.	Bandwidth (GHz)	Isolation Level (dB)	ECC Value	Size (λ_0_ × λ_0_)	Number of Radiating Elements
[13]	24.1–27.2	16	0.1	2.08 × 1.73	2
[14]	3.4–3.6	15	0.16	1.43 × 0.85	4
[16]	3.4–3.6	18.4	0.08	1.2 × 1.2	8
[21]	3.4–3.6	20	0.06	1.75 × 0.88	4
[22]	3.4–3.6	13	0.1	1.63 × 0.82	8
[23]	3.6–9.8	17.3	0.04	1.06 × 0.75	4
[24]	2–3	8	0.25	0.57 × 0.57	2
This Work	2.43–2.48	20	0.01	1.22 × 0.79	6

**Table 2:** The performance for the three cases.

Case Number	Signal (Azimuthal, Elevation) Angles	Interference (Azimuthal, Elevation) Angles	Array Size	Interference Peak Power without Beamforming in dBm	Interference Peak Power with Beamforming in dBm
1	(45°, 45°)	(30°, 90°)	3 × 2	−26	−38
2	(30°, 45°)	(45°, 70°)	3 × 2	−26	−28
3	(30°, 45°)	(45°, 70°)	3 × 4	−27	Almost removed

## Data Availability

Not applicable.
